# Transparent, abrasion-insensitive superhydrophobic coatings for real-world applications

**DOI:** 10.1038/s41598-017-15287-8

**Published:** 2017-11-08

**Authors:** Dorothea Helmer, Nico Keller, Frederik Kotz, Friederike Stolz, Christian Greiner, Tobias M. Nargang, Kai Sachsenheimer, Bastian E. Rapp

**Affiliations:** 1 Institute of Microstructure Technology (IMT), Karlsruhe Institute of Technology (KIT), Hermann-von-Helmholtz-Platz 1, 76344 Eggenstein-Leopoldshafen, Germany; 2 Institute for Applied Materials - Computational Materials Science (IAM-CMS), Karlsruhe Institute of Technology (KIT), Engelbert-Arnold-Str. 4, 76131 Karlsruhe, Germany

## Abstract

Superhydrophobic surfaces and surface coatings are of high interest for many applications in everyday life including non-wetting and low-friction coatings as well as functional clothing. Manufacturing of these surfaces is intricate since superhydrophobicity requires structuring of surfaces on a nano- to microscale. This delicate surface structuring makes most superhydrophobic surfaces very sensitive to abrasion and renders them impractical for real-life applications. In this paper we present a transparent fluorinated polymer foam that is synthesized by a simple one-step photoinitiated radical polymerization. We term this material “Fluoropor”. It possesses an inherent nano-/microstructure throughout the whole bulk material and is thus insensitive to abrasion as its superhydrophobic properties are not merely due to a thin-layer surface-effect. Due to its foam-like structure with pore sizes below the wavelength of visible light Fluoropor appears optically transparent. We determined contact angles, surface energy, wear resistance and Vickers hardness to highlight Fluoropor’s applicability for real-word applications.

## Introduction

Transparent, superhydrophobic surfaces are of high interest especially for functional coatings. In order to achieve superhydrophobic properties, i.e. contact angles greater than 150° and roll-off angles of less than 10°^1,2^, the surface must be engineered in terms of chemistry and surface roughness: only appropriately structured surfaces achieve the superhydrophobic state. State-of-the art techniques for generation of transparent, superhydrophobic surfaces rely on a) deposition or growth of functionalized or embedded nanoparticles (mostly spheres or rods) on surfaces^3–6^, b) custom-engineered surface structures (pillars) or induced surface roughness by etching^7,8^, anodization^9^ or imprinting^10^ with optional consecutive hydrophobic functionalization and c) phase-separation effects in polymer deposition upon drying, heating or during polymerization^11,12^. Most of the reported methods yield thin and fragile coatings which have limited practical relevance mostly due to their abrasion-sensitivity. Thick-layer coatings with nano-/microstructuring throughout the bulk material however would circumvent the problem of abrasion-sensitivity: all layers exposed through abrasion effects display superhydrophobicity due to their inherent structure. Bulk porous polymers with superhydrophobic properties have been reported^13–17^, as well as self-healing materials based on silica powders^18–20^, however none of the materials combine thick-layer coatings with transparency and abrasion-insensitivity and most reported foams possess low stability owing to their aerogel-like structure.

Here we describe an easy-to-fabricate, optically transparent, abrasion-insensitive bulk nano-/microstructured polymer that can be used for coating surfaces (glass, metals and textiles) as well as bulk substrates. We call this material “Fluoropor”. Fluoropor is made via radical polymerization in a one-step procedure with foam-like structuring just below the threshold of light scattering resulting in optical transparency of the material. All components of Fluoropor are readily commercially available. We have characterized Fluoropor for its real-world applicability by standardized methods such as wear resistance and Vickers hardness measurements which are employed for coating characterization on an industrial scale^21^. Fluoropor is insensitive even to dramatic abrasion such as scratching or sandpaper abrasion making it ideal for long-term use under harsh abrasive conditions. Fluoropor is an easy-to-create, widely-applicable and wear/abrasion-tolerant material for real-world applications.

**Figure 1:**
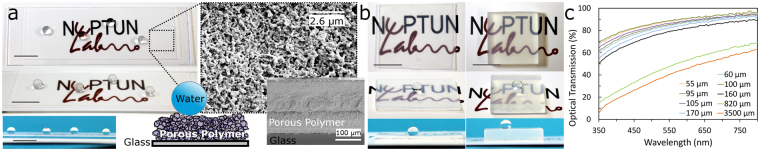
Transparent, bulk nano-/microstructured polymer foam “Fluoropor”. (**a**) Thin-layer (105 µm) optically transparent Fluoropor film on glass with superhydrophobic properties. The SEM images show the porous nano-/microstructure of the material on the surface and in the bulk (side view of a 210 µm coating on glass). (**b**) Fluoropor bulk nano-/microstructured samples of 820 µm and 3500 µm thickness. (**c**) Optical transmission of Fluoropor samples with varying average thicknesses between 55 µm and 3500 µm. All scale bars: 1 cm. Optical transmissions were measured against a blank of the supporting glass and therefore display the values for the coating only.

We achieved the formation of superhydrophobic transparent polymer foam “Fluoropor” through light-induced polymerization of fluorinated perfluoropolyether methacrylates in a non-solvent (cyclohexanol) and an emulsifying agent (fluorinated alcohol). Fluoropor possesses a bulk nano-/microstructure (see Fig. 1a and Supplementary Fig. S1) and can be polymerized in varying thicknesses (see Fig. 1b). Due to the polymerization process Fluoropor samples possess a non-structured layer of polymer on top (“lid” see Supplementary Fig. S2) which can be readily removed by abrasion. The optical transparency of Fluoropor is mainly dependent on the degree of light scattering of the surface exposed by the removal of the lid. Thus there is no linear correlation between layer thickness and optical transparency. All thin-layer glass coatings between 55 µm and 170 µm show high optical transparency within the visible light region (see Fig. 1c). Bulk samples of 800 µm and 3500 µm thickness show decreased light transmission but are still see-through upon background contact (see Fig. 1b,c). The transparency of foams is dependent on light scattering at the pore surfaces via reflection and refraction^22^. When the pores are small enough, i.e. when their diameter is well below the wavelength of visible light, the material appears transparent. At 400 nm, non-foamed Fluoropor possesses an optical transmission of 99%, foamed Fluoropor with a smooth surface (lid structure intact) possesses an optical transmission of 86% and the lowest transmission is displayed by foamed Fluoropor with a rough surface (lid structure removed) with 66% at 400 nm (100 µm films, see Supplementary Fig. S3). Therefore, as expected, the pore size in the bulk as well as the surface roughness are responsible for the loss in transparency. Still the pores are small enough to make Fluoropor appear transparent. Optical transmissions were measured against a blank of the supporting glass and therefore display the values for the coating only. The transmission of the supporting glass is displayed Supplementary Fig. S4. Functionalization of glass surfaces relies on exposing the surface silanol groups by treatment with methanol/hydrochloric acid (1:1 v/v)^23^ and consecutive attachment of 3-(dimethylchlorosilyl)propyl methacrylate to the surface. The methacrylate groups are covalently attached to the glass surface and take part in the polymerization reaction of Fluoropor thus covalently attaching the polymer.

**Figure 2:**
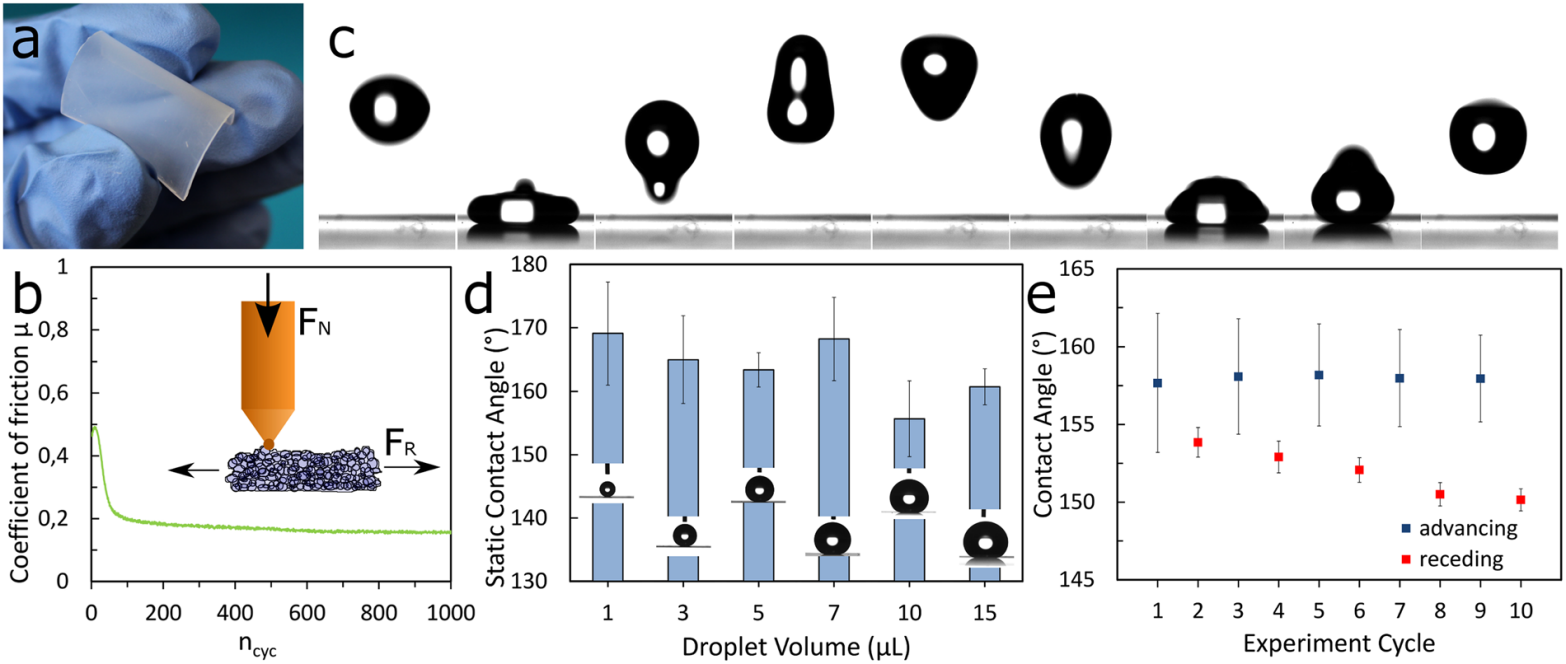
Characterization of Fluoropor bulk nano-/microstructured polymer foam. (**a**) Fluoropor is a soft material and a substrate of 820 µm thickness is very flexible. (**b**) The wear resistance of Fluoropor was measured using a tribometer (schematic inset). After a break-in phase of ~50 cycles, a coefficient of friction of µ = 0.2 was determined (µ = F_R_/F_N_). (**c)** A 5 µL droplet of water bounces off a Fluoropor surface (time passed between frame 1 and frame 9: approximately 2.6 s). (**d**) The static contact angle of Fluoropor shows little variation at various droplet volumes and was determined to be an average of 163.7 ± 6.8°. (**e**) Advancing and receding contact angle measurements of Fluoropor gave 158.0 ± 7.9° and 151.9 ± 1.9°, respectively. The receding contact angle decreases due to evaporation of the droplet over time.

Fluoropor possesses a low Vickers hardness of 1.19 HV at 100 mN/20 s – which is in the appropriate range of rigid polymer foams^24^. For further comparison, polyethylene possesses a Vickers hardness of 5–8 HV^24^. Due to its softness Fluoropor is very flexible at layer thicknesses below ~1000 µm (see Fig. 2a). Polymers and fluoropolymers in particular are of high interest for low-friction coatings, i. e. coatings with friction coefficients of 0.4 and lower^25^. Wear resistance measurements of Fluoropor gave a coefficient of friction of µ = 0.2 sliding against a brass sphere (see Fig. 2b) which is comparable to polytetrafluoroethylene (PTFE) sliding against stainless steel (µ = 0.15–0.2)^26^. The wear tracks of the tribometer were below 1 µm in depth thus the material is sufficiently robust for prolonged wear-abrasion (see Supplementary Fig. S5). Fluoropor was further characterized in terms of its superhydrophobic properties. A droplet of water readily bounces off a Fluoropor surface (see Fig. 2c). The superhydrophobicity is independent on the drop volume when gravitational effects on the drop-shape are minor, i.e. between 1 µL and 15 µL and the average static contact angle of Fluoropor was calculated to be 163.7 ± 6.8° (see Fig. 2d). The static contact angle is a common but not an ideal characterization method for rough surfaces. The maximum (advancing) and minimum (receding) contact angles are better suited for producing reliable results on inhomogeneous surfaces. The advancing and receding contact angles were determined to be 158.0 ± 7.9° and 151.9 ± 1.9°, respectively (see Fig. 2e) with an average contact angle hysteresis of 6.1°. The variations in the advancing contact angle are due to vibrations of the enlarging droplet during the measurement (see Supplementary Fig. S6), which lead to variations in the contact angles determined by the instrument automatically by evaluating the high-speed video data image-by-image. The decrease in receding contact angles over time is caused by decreasing contact radius of the droplet due to evaporation over time^27^. The surface possesses a very low free energy of 7.2 mN/m. In accordance with the low hysteresis a water roll-off-angle of 6.6 ± 1.7° was determined.

**Figure 3:**
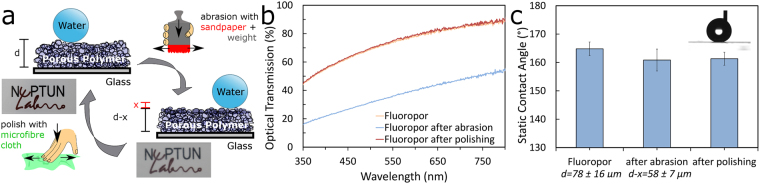
The superhydrophobic properties of Fluoropor are insensitive to abrasion. (**a**) Schematic illustration of Fluoropor abrasion-test: Fluoropor sample of thickness *d* [µm] is abraded by *x* [µm] by using sandpaper under a weight (0.74 kg). The superhydrophobic properties of the surface are retained due to its bulk porosity, while the transparency of the polymer suffers lightly (see photographic insets). Transparency is regained by polishing with a microfiber cloth. (**b**) Optical Transmission of the Fluoropor coating before abrasion, after abrasion of *x*~20 µm and after polishing. The optical transparency of the coating is fully regained after sandpaper abrasion and consecutive polishing. (**c**) Static contact angles of water on Fluoropor before abrasion, after abrasion and after polishing remain nearly constant, making the superhydrophobic properties of Fluoropor insensitive even to drastic abrasion of 20 µm layer thickness.

Abrasion is a threat to any functional structured coating. Fluoropor with its high wear-resistance is not easily abraded but if abrasion occurs the inherent bulk nano-/microstructure makes its superhydrophobic properties insensitive to the abrasion (see Fig. 3). Upon treating Fluoropor with sandpaper and abrading a total of 20 µm of the ~80 µm coating the superhydrophobic properties of the surface are fully retained with a minor change in the static contact angle from 165 ± 2° to 161 ± 4°. Scratching the surface leads to slightly decreased transparency (see Fig. 3b), however the transparency of the material can be recovered by polishing the surface with a microfiber cloth which has no influence on the contact angle which was determined to be 161 ± 2° after polishing (see Fig. 3c). SEM images of Fluoropor after abrasion and after abrasion and consecutive polishing (see Supplementary Fig. S7) show the unaltered nano-/microstructure of Fluoropor.

**Figure 4:**
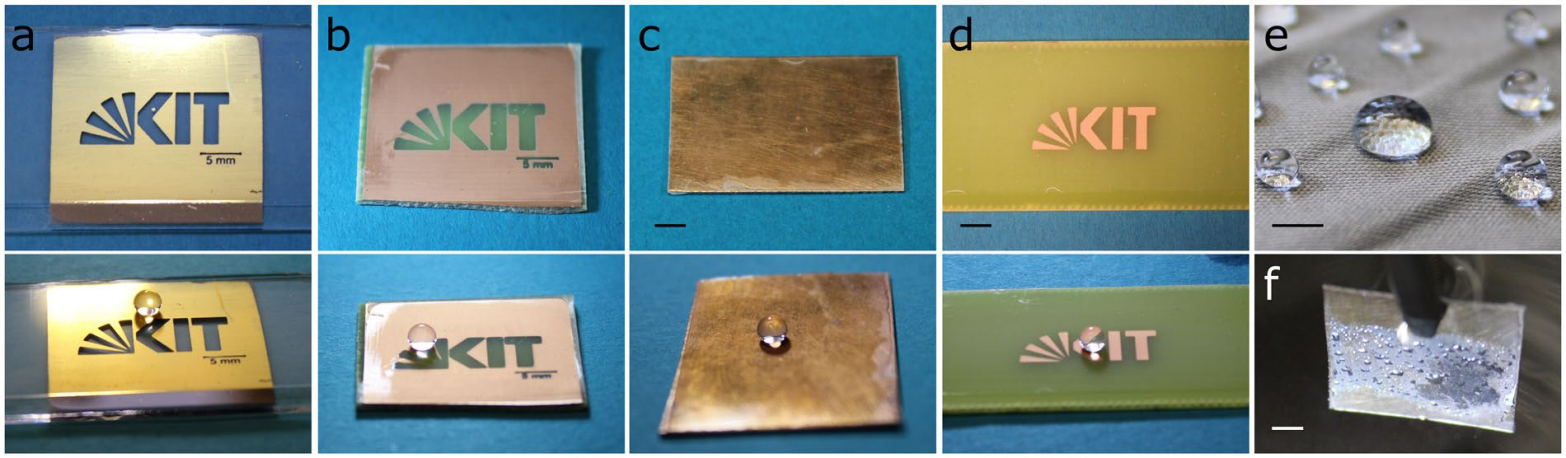
Fluoropor coatings on metal, polymer and textile surfaces. Images above: top view of the substrates, images below: side view with 20 µL droplets of water on the superhydrophobic surfaces. (**a**) Fluoropor on gold (glass slide with chromium and gold layer, partially etched), static contact angle: 153.4 ± 4.6°. (**b**) Fluoropor on copper (circuit board partially etched), static contact angle: 153.1 ± 1.6°. (**c**) Fluoropor on copper sheet, static contact angle: 157.9 ± 2.0°, scale bar: 5 mm. (**d**) Fluoropor on an epoxy polymer (circuit board, partially etched), static contact angle: 154.9 ± 2.5°, scale bar: 5 mm. (**e**) Fluoropor on microfiber cloth, static contact angle: 148.9 ± 1.1°, scale bar: 5 mm. (**f**) Fluoropor on copper sheet shown in **c** forms an air-retaining *Salvinia* layer under water.

Besides glass, Fluoropor was also coated on other practically relevant surfaces such as metals, polymers and textiles to show its applicability as a superhydrophobic coating (see Fig. 4). Fluoropor films in the range of ~100 µm were polymerized onto gold, copper and epoxy surfaces using a supporting layer of silane. The metal surfaces were functionalized with a protocol adapted from Wu *et al*.^28^ where a self-assembled monolayer of 3-(mercaptopropyl) triethoxysilane is formed on the metal surface, followed by hydrolysis and condensation of the triethoxysilane groups by corona discharge to form a siloxane network with exposed silanol groups. In a second step, the silanol groups react with 3-(dimethylchlorosilyl)propyl methacrylate to give surface-bound methacrylate groups that covalently attach Fluoropor during polymerization. Fluoropor can also be used as a coating for textiles (see Fig. 4e) coating the fibers with a layer of polymer (see Supplementary Fig. S8). Under water the coated substrates form an air-retaining *Salvinia* layer (see Fig. 4f). Fluoropor on copper was further employed to demonstrate the triboelectrical properties of Fluoropor: rubbing a copper sheet against a copper sheet coated with Fluoropor gave a voltage of 3.5 V.

We demonstrated the formation of nano-/microstructured polymer foam “Fluoropor” with adjustable thickness and high optical transparency. The abrasion-insensitivity of the superhydrophobic properties of Fluoropor, as well as its ease of manufacturing and its compatibility with a wide range of substrate materials makes Fluoropor a transparent coating suitable for real-world applications.

## Methods

Fluorolink MD700 was purchased from Acota (United Kingdom), 3-(methacryloyloxypropyl) dimethylchlorosilane, 3-(mercaptopropyl)triethoxysilane, methanol, ethanol, methacrylic anhydride, toluene, cyclohexanol, acetone and 2-propanol, hydrochloric acid (37%) were purchased from Merck (Germany). 1 H,1 H,2 H,2H-perfluorooctanol was purchased from Apollo Scientific (United Kingdom) and 2,2-dimethoxy-2-phenylacetophenone (DMPAP) was purchased from Sigma-Aldrich (Germany). Nexterion Slide Glass B clean room slides were purchased from Schott (Germany). AZ photoresist and developer were purchased from microchemicals (Germany).

### Synthesis of nano-/microstructured transparent fluoropolymer

50 wt% Fluorolink MD700 was mixed with 15 wt% cyclohexanol and 35 wt% 1 H,1 H,2 H,2H-perfluorooctanol. The mixture was blended with 0.5 wt% DMPAP dissolved in acetone (1 mg/µL). Custom-made polymerization chambers were constructed from regular glass slides, glue and spacers of different height (made from cyclic olefin copolymer, COC). The mixture was polymerized under UV light (370 nm, Lumatec Superlite SUV mercury arc lamp, Lumatech, Germany) for 2–5 min depending on the layer thickness. The polymer was placed in 2-propanol overnight and consecutively dried in a vacuum furnace at 100 °C for 1 h at 30 mbar. The top layer of the Fluoropor samples was removed after drying to expose the nanostructure due to the polymerization of a non-structured layer on top of the polymerization chamber (see Supplementary Fig. S2). This layer can be removed by regular laboratory cotton wipes in case of thin-layer coatings or by abrasion with sandpaper for thicker substrates.

### Functionalization of glass slides

Cleanroom glass slides were immersed in a mixture of concentrated hydrochloric acid and methanol (50:50) for 30 min. Afterwards the slides were rinsed with 2-propanol and deionized water and dried with compressed air. To functionalize the surface, the slides were incubated in a 100 mM solution of (3-methacryloyloxypropyl)dimethylchlorosilane in toluene for 1 h. The slides were rinsed with 2-propanol and deionized water and dried with compressed air.

### Functionalization of copper, gold and epoxy substrates

Gold slides and copper slides were etched to show the KIT logo by etching with iodine solution and ammonium persulfate solution, respectively, using AZ1514H photoresist as a mask. The metal substrates were cleaned with 2-propanol and dried with compressed air. The substrates were placed in 2 vol% solution of 3-(mercaptopropyl)triethoxysilane in ethanol for 8 min. The substrates were rinsed with deionized water and dried with compressed air. After cleaning the metal substrate were activated with a corona discharger (type BD 20, purchased from ETP, USA) for about 1 min. During activation the corona discharger was placed at a distance of approximately 0.5 cm above the sample surface. The substrates were then immersed in a 100 mM solution of 3-(dimethylchlorosilyl)propyl methacrylate in toluene for 1 h. The slides were rinsed with 2-propanol and deionized water and dried with compressed air.

### Textile functionalization

The microfiber cloth was used without prior treatment.

### Contact angle measurement

Contact angles were measured with a OCA15 Pro (Data Physics, USA). All droplet-shapes were evaluated by using the Young-Laplace fit if not stated otherwise. For advancing and receding contact angle measurements by the ARCA program of the instrument, a 5 µL droplet of deionized water was dispensed onto the surface and held in place by the dispenser needle. Additional 5 µL of deionized water were dispensed to a final droplet volume of 10 µL (advancing contact angle). After 1 min 5 µL of the droplet volume were removed (receding contact angle). After 1 min the droplet was expanded to 10 µL and consecutively after 1 min reduced to 5 µL again. In this way five receding and five advancing contact angles were measured by evaluation of the video data, in this case the contact angles were determined by the tangent-fit to reduce computing time. Left- and right-sided contact angles were determined for each frame and the arithmetic middle of the two angles was plotted. The roll-off angle was characterized by dispensing a 10 µL droplet onto the surface and consecutive tilting of the instrument at a given angle per time. Static contact angles and the surface energy of the material were determined using 5 µL droplets. For the determination of the free surface energy, the static contact angles of 5 µL droplets of deionized water and diiodomethane were determined and the OWRK method was used to calculate the surface energy.

### UV/VIS measurements

The optical transmission of the nano-/microstructured Fluoropor samples was determined using a UV/VIS spectrometer (Evolution 201, Thermo Scientific, Germany). Coatings on glass were measured against a glass blank, free polymer substrates were measured against air.

### Vickers Hardness

The Vickers hardness was determined using a Vickers hardness tester (Fischerscope HCU, Fischer, Germany) at a load of 100 mN and a loading time of 20 s.

### Wear Resistance

The wear resistance was determined using a custom-built tribometer equipped with a brass test body of 8 mm diameter at 50% relative humidity (RH). The test body moves across the sample thereby causing abrasion. Samples were tested with a load of 400 g, a glide path of 10–12 mm, v = 0.5 mm/s and 1000 experimental cycles.

### Sandpaper abrasion test

Fluoropor coatings on glass were glued to the table using double-sided adhesive tape. 2500 grain sandpaper was glued onto the bottom of a flat 0.74 kg Teflon block. The block was moved across the sample approximately 100 times to give an even abrasion across the whole sample. The sample was cleaned by careful strokes with a cotton wipe, microfiber cloth and/or usage of a pressurized air gun to ensure the full removal of any fluorinated particles.

### Layer thickness measurements

The thicknesses of thin-layer coatings on glass were determined using a Heidenhain MT 60 M length gauge. Thicker unsupported samples were measured using a caliper.

### Triboelectric measurement

A ~ 100 µm layer of Fluoropor was coated onto a copper plate. A second copper plate was rubbed against the coated plate. Both plates were connected to a multimeter to determine the voltage produced by rubbing.

### Data availability statement

The datasets generated during and/or analysed during the current study are available from the corresponding author on reasonable request.

## Electronic supplementary material

Supplementary Information

M1

M2
