# Supplementary material for: Transparent, abrasion-insensitive superhydrophobic coatings for real-world applications
Source: Sci Rep. 2017 Nov 8;7:15078. doi: 10.1038/s41598-017-15287-8 (PMC5678135; doi:10.1038/s41598-017-15287-8)
Supplement: Supplementary file 1 — Supplementary Information [file 41598_2017_15287_MOESM1_ESM.doc]

Supplementary Information

Transparent, abrasion-insensitive superhydrophobic coatings for real-world applications

Dorothea Helmer^1†^, Nico Keller^1†^, Frederik Kotz^1^, Friederike Stolz^2^, Christian Greiner^2^, Tobias M. Nargang^1^, Kai Sachsenheimer^1^, Bastian E. Rapp^1^*

*† Contributed equally to this work.*

^1^Institute of Microstructure Technology (IMT), Karlsruhe Institute of Technology (KIT), Hermann-von-Helmholtz-Platz 1, 76344 Eggenstein-Leopoldshafen, Germany.

^2^Institute for Applied Materials - Computational Materials Science (IAM-CMS), Karlsruhe Institute of Technology (KIT), Engelbert-Arnold-Str. 4, 76131 Karlsruhe, Germany.

*Correspondence to: bastian.rapp@kit.edu.

Supplementary Materials

**Supplementary Videos**

Movie M1: Water droplet jumping on Fluoropor

Movie M2: ARCA advancing /receding contact angle measurement


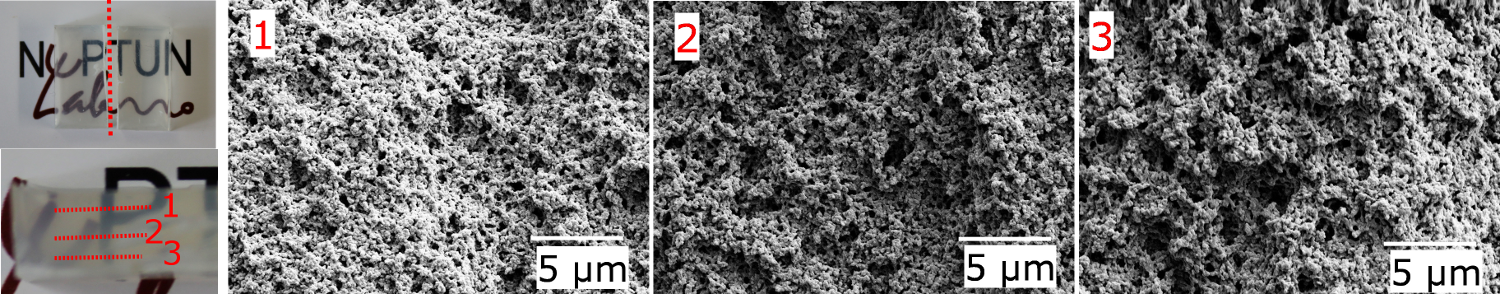


Figure S1 | Bulk nano /microstructured ~3500 µm thick Fluoropor substrate. The Fluoropor sample was broken in the middle (red dotted line) and the broken edge was imaged at three different points throughout the bulk (red dotted lines 1-3) using SEM.


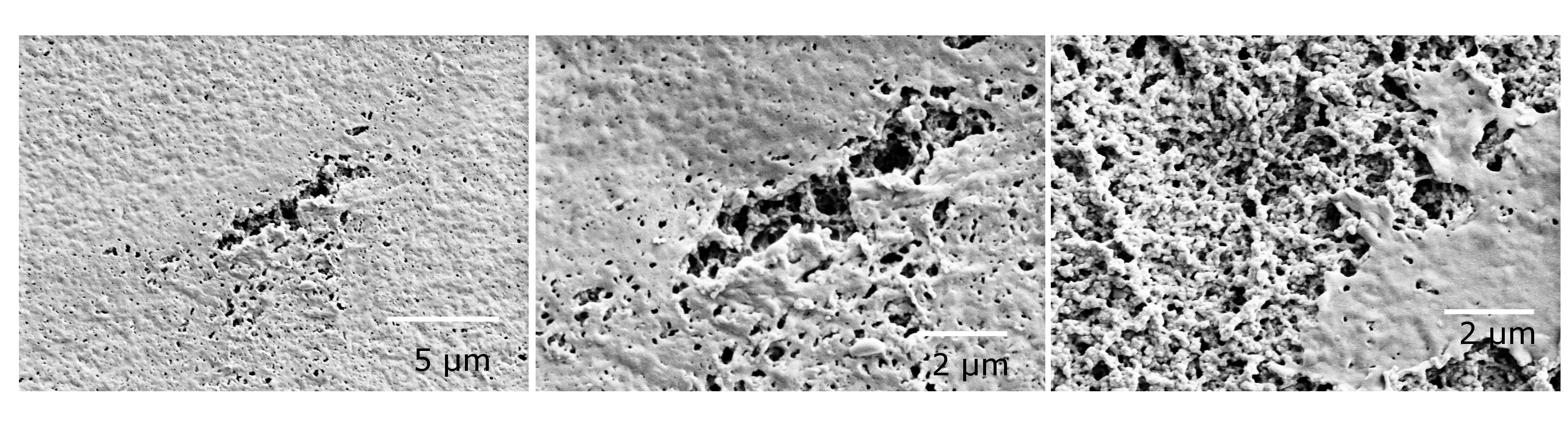


Figure S2 | Lid structures of Fluoropor substrates. Polymerization in a closed chamber leads to the formation of a non-structured layer on top of the substrate. The nano /microstructure underneath the non-structured layer is clearly visible.


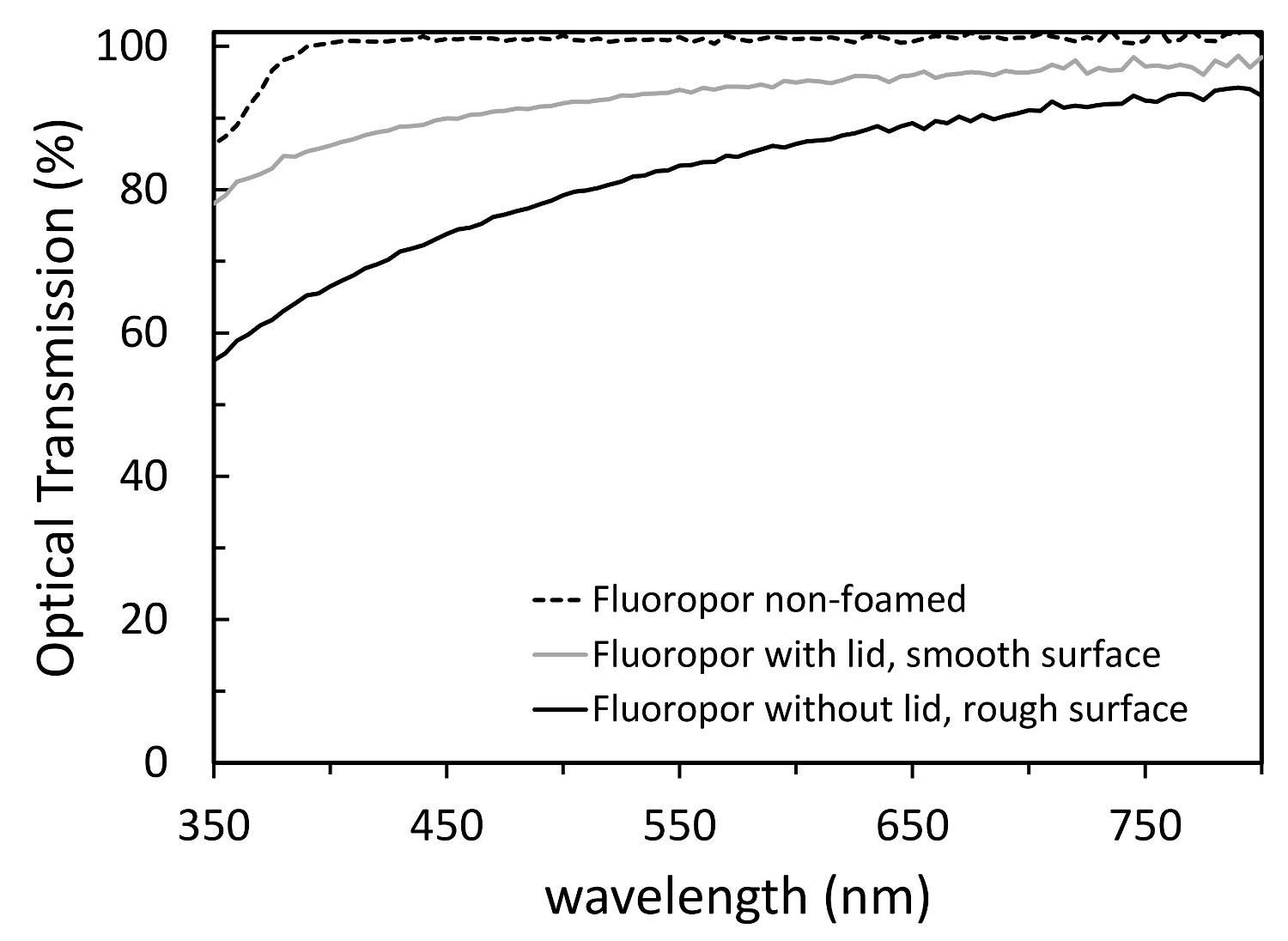


**Figure S3 | Influence of foam cavities and surface structure of Fluoropor on the optical transmission of the material.** 100 µm films on glass were measured against an equivalent glass blank. At 400 nm non-foamed material (MD700) shows an optical transmission of 99 %, foamed Fluoropor with an intact lid structure (smooth surface, see also Figure S2) shows an optical transmission of 86 % and foamed Fluoropor where the lid structure has been removed (rough surface) shows an optical transmission of 66 %. Scattering at the pore surfaces inside the material therefore has an influence on the transparency of Fluoropor as well as scattering at the surface structures. The decrease of optical transmission of MD700 around 390 nm is a result of residual photoinitiator byproducts and the general yellowish colour of the commercial MD700. This transmission can be optimized by immersing polymerized MD700 in polar solvents for several hours. The production process of Fluoropor, where MD700 is mixed with solvents which are later removed, also removes the coloring and the residual photoinitiator byproducts in the course of processing, which is why no drop in transmission is observed for foamed Fluoropor.


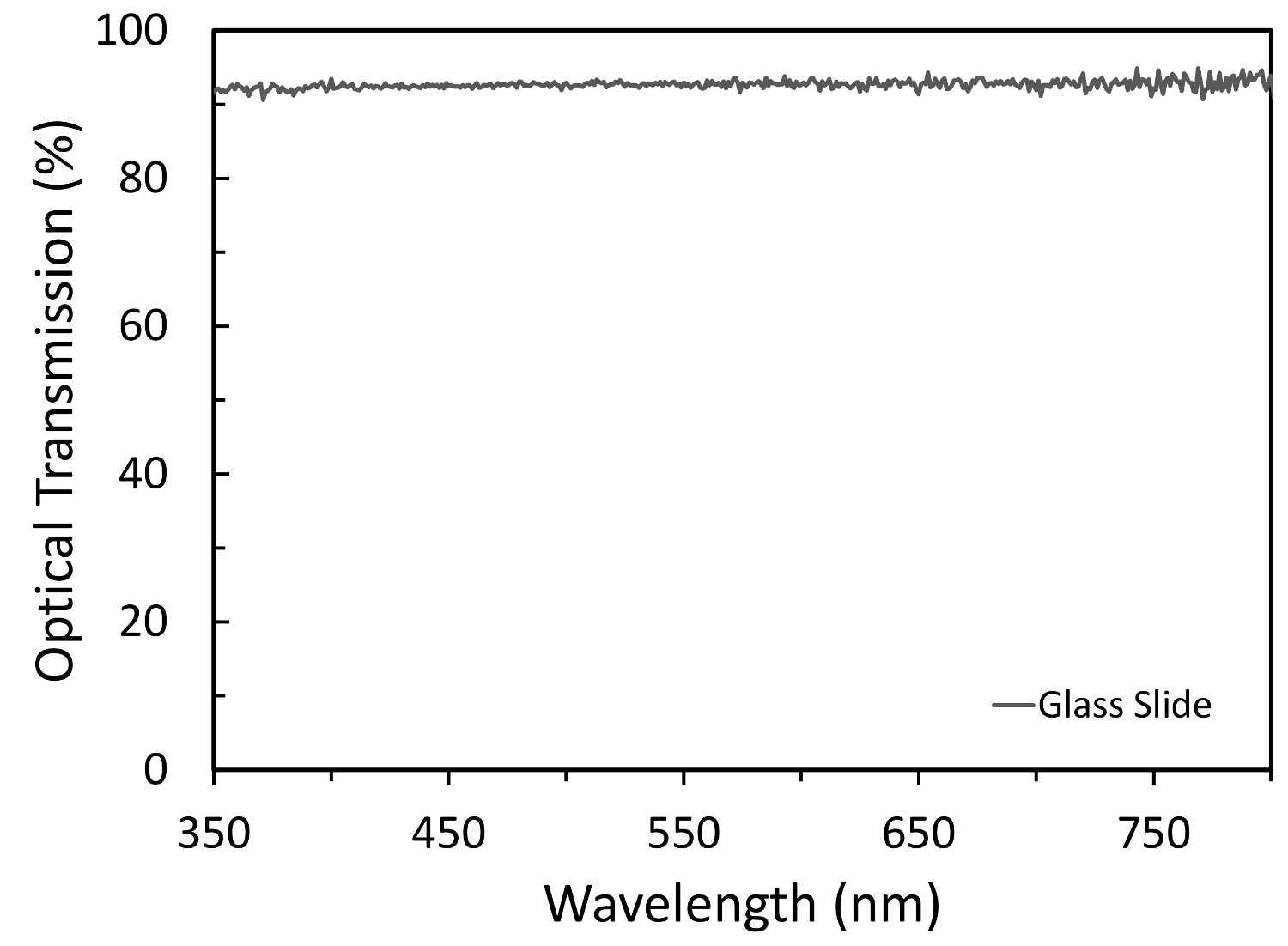


**Figure S4 | Transparency of the glass slides used as support for the coatings of Fluoropor.** Schott Nexterion Slide Glass B clean room slides were used for coating, all UV/VIS measurements on glass slides were measured against a blank measurement of these slides.


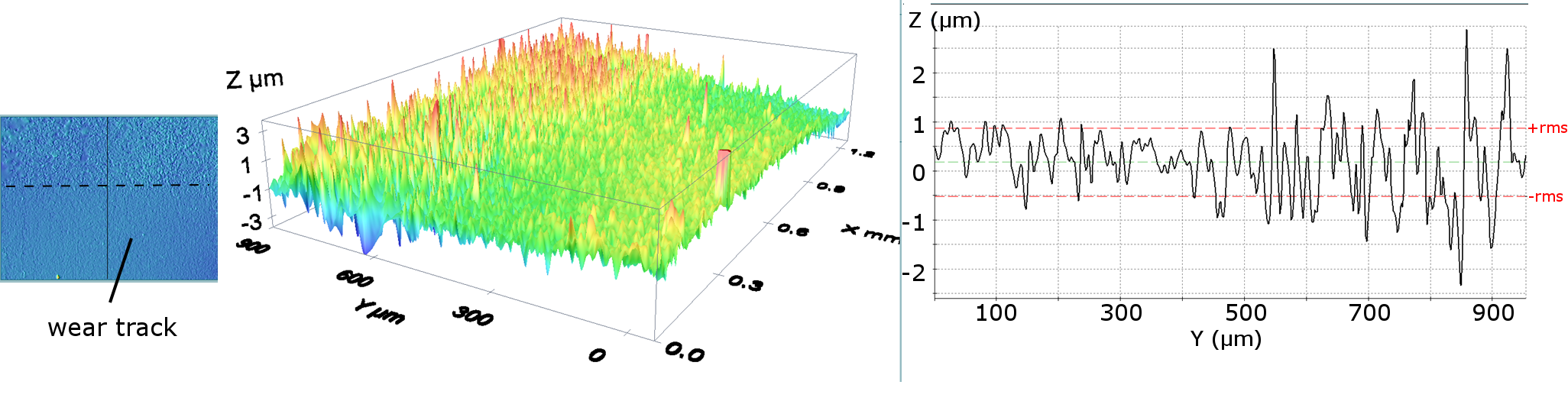


Figure S5 | Surface topography of Fluoropor after wear abrasion test with a tribometer. The wear tracks are below 1 µm showing the robustness of the material.


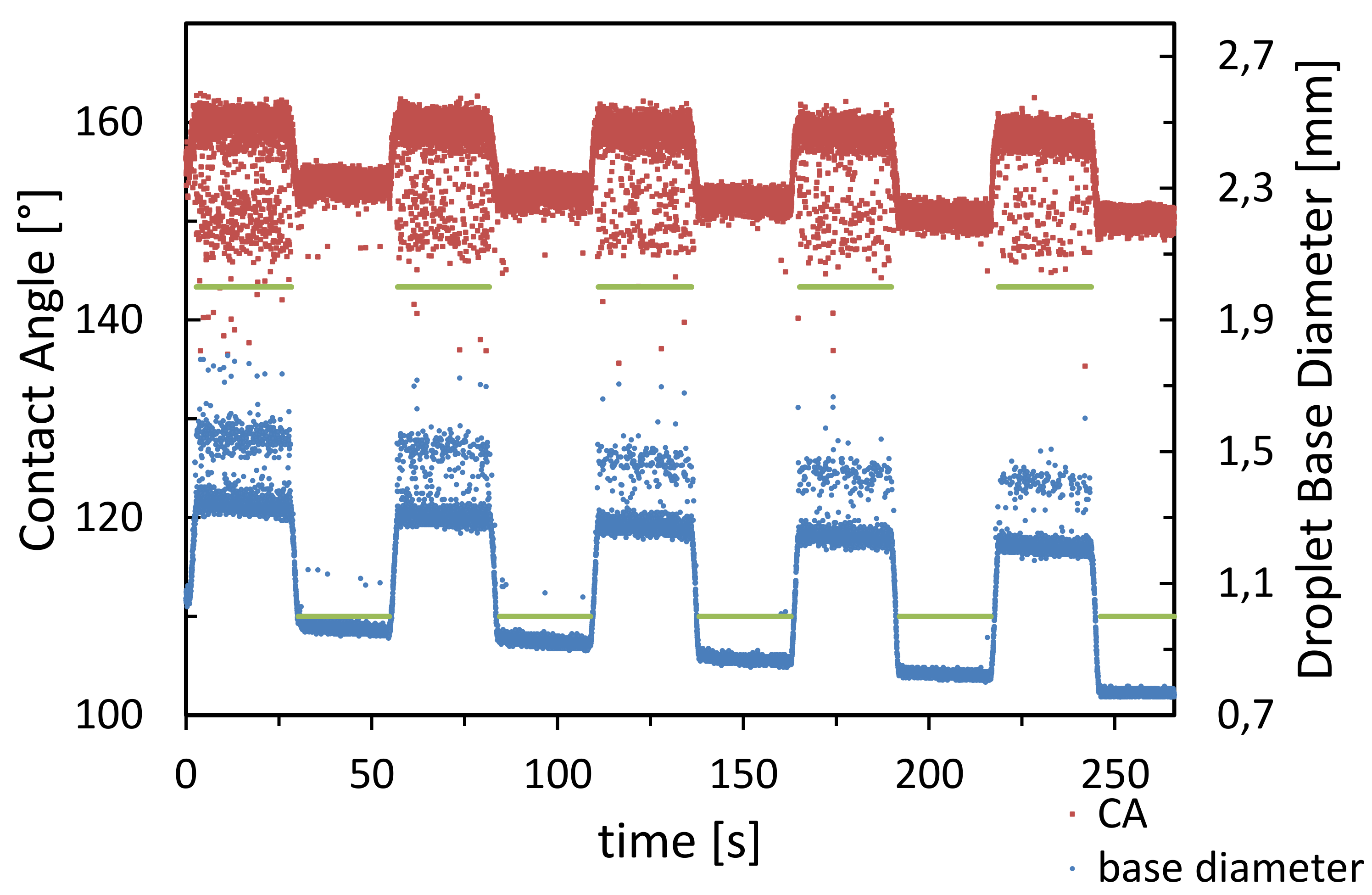


Figure S6 | Full exemplary advancing/receding contact angle measurement conducted by the ARCA method of the OCA 15pro (tangent-fit for determination of the contact angles). The droplet size diminishes due to evaporation (blue). The advancing and receding contact angles were determined by calculating the average contact angle on the plateaus (timepoints indicated by green lines). Variations in the contact angle measurements are due to the high contact angles determined by the tangent-fit and the vibrations of the droplet, see Video M2.


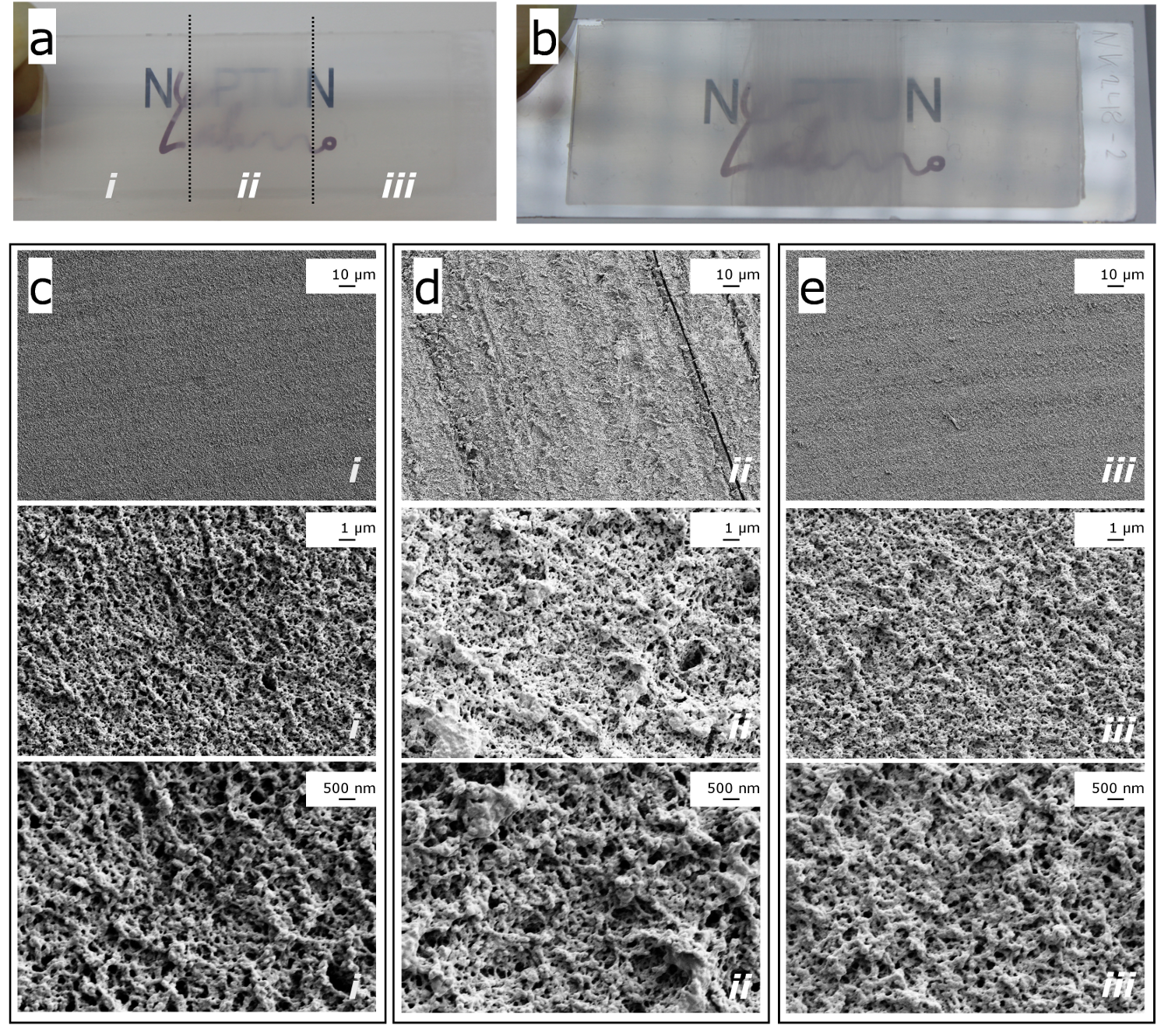


Figure S7 | SEM images of Fluoropor before abrasion, after abrasion and after polishing. a, Fluoropor on glass without lid (i), abraded with sandpaper (ii) and polished with a microfiber cloth after abrasion with sandpaper (iii). b, For enhanced visibility of the three areas of the slide shown in a, the slide was slightly tilted towards the light source. c, SEM images of three magnifications of area *i* of the slide shown in a: Fluoropor without lid. d, SEM images of three magnifications of area *ii* of the slide shown in a: Fluoropor abraded with sandpaper. This area shows notable scratch marks from the sandpaper at low magnification. e, SEM images of three magnifications of are iii of the slide shown in a: Fluoropor abraded with sandpaper and consecutively polished with a microfiber cloth. This area shows scratch marks which are less pronounced. At high magnifications all areas show the same, characteristic nano-/microstructuring of Fluoropor.


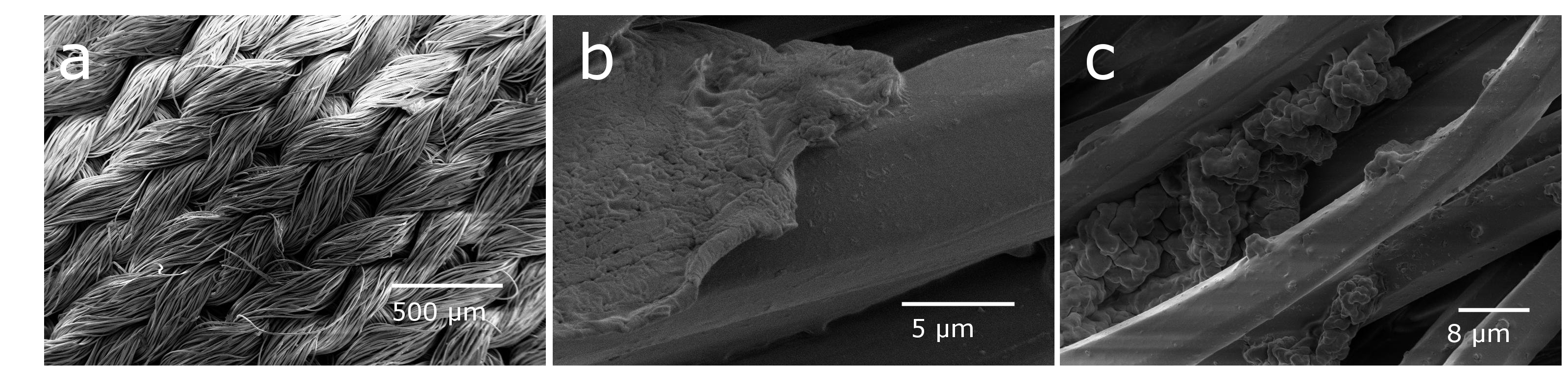


Figure S8 | Microfiber fabric with Fluoropor coating. The fibers are covered in fluoropolymer with inherent porosity.
